# Impact of Intravenous Tranexamic Acid on Hemorrhage During Endoscopic Sinus Surgery 

**Published:** 2015-09

**Authors:** Saeedollah Nuhi, Ali Goljanian Tabrizi, Leyla Zarkhah, Bahram Rashedi Ashrafi

**Affiliations:** 1 *Department of Otorhinolaryngology, Shahid Beheshti University of Medical Sciences, Taleghani Hospital, Tehran, Iran.*

**Keywords:** Endoscopic sinus surgery, Hemorrhage, Tranexamic acid

## Abstract

**Introduction:**

Endoscopic sinus surgery is a common procedure performed by otolaryngologists. This study evaluated the efficacy of intravenous (IV) tranexamic acid (TA) on hemorrhage in patients undergoing elective endoscopic sinus surgery (ESS).

**Materials and Methods:**

The present study was performed in 170 patients scheduled for ESS surgery under general anesthesia in order to examine the effects of IV TA on providing a bloodless surgical field and to evaluate the amount of bleeding. One hundred patients received intravenous TA and 70 patients received placebo. Intraoperative hemorrhage was estimated by the attending anesthesiologist at the end of surgery by accounting for loss of blood and irrigation fluid in a 25 mL-graded suction canister and nasopharyngeal packing (measured weight of packing on the electronic scale). Hemodynamic variables were monitored and coagulation profile was determined.

**Results:**

A total of 170 patients (90 male [53%] and 80 female [47%]), mean age 30.54±4.14 years, were evaluated. There was a significantly lower bleeding volume in the TA group than in the placebo group (107.7±45.1 vs. 189.3±51 mL; P<0.001). There was no significant difference between pre- and postoperative hematocrit (38.81± 4.20 vs. 36.60± 3.35) or pre- and postoperative hemoglobin (12.51± 2.5 vs. 11.64±1.9) levels in the TA group (P>0.05). Moreover, the difference between the TA and control groups regarding postoperative hematocrit (34.65±4.45 vs. 36.60±3.35) and hemoglobin (10.81±2.1vs. 11.64±1.9) levels was not significant (P>0.05). Vomiting and nausea in the control group was greater than in the control group, but the difference was not significant (P>0.05). We did not detect significant coagulation alterations in the TA group.

**Conclusion:**

TA significantly decreased hemorrhage without increasing side effects such as alteration in coagulation parameters, hemodynamic changes, and vomiting and nausea. Use of TA can avoid the need for antihypertensive agents to reduce blood loss in ESS.

## Introduction

Endoscopic sinus surgery (ESS) is the most common procedure performed by otolaryngologists, because of its high success rate, low incidence of complications, advances in instrumentation and imaging, and the introduction of computer-aided surgery (1,2). 

However,hemorrhage during and following ESS is an issue of concern for surgeons, and reduction of hemorrhage is an important challenge (3). Earlier studies have confirmed the favorable effects of tranexamic acid (TA), on bleeding tendency in patients undergoing surgery (4,5). Techniques such as bipolar diathermy, packing, topical vasoconstrictors, and induced hypotension have been used to improve the surgical field in ESS; however a number of complications are associated with these methods. For example, diathermy can cause local tissue damage and subsequent bleeding, and topical vasoconstrictors can cause hemodynamic instability, especially in patients with a history of hypertension or ischemic heart disease. Furthermore, induced hypotension exposes patients to more anesthetic drugs and their associated side effects. In addition, none of these drugs consistently provides a desirable bloodless field for surgeons (6–8).

TA is an antifibrinolytic agent that blocks lysine binding sites on plasminogen, thereby inhibiting the interaction of plasminogen and the heavy chain of plasmin with lysine residues on the surface of fibrin (9). Some studies have reported the efficacy of topical and oral forms of TA in achieving hemostasis and improving the surgical field in nasal surgery including functional endoscopic sinus surgery (FESS) (10,11). Furthermore, Senghore and Harris revealed that intravenous (IV) administration of TA is effective in preventing excessive postoperative bleeding in healthy adult patients undergoing dental surgery (12). In this study,the efficacy of IV TA in reducing bleeding associated with nasal surgery (i.e; FESS) was examined.

## Materials and Methods

In this controlled, double-blind clinical trial, 170 patients scheduled for elective ESS because of chronic sinusitis were enrolled from 2009 to 2011.The study protocol was approved by the Shahid Beheshti University of Medical Sciences Ethics Committee. Furthermore, the study procedure was explained to all patients, and written, informed consent was obtained. The exclusion criteria were presence of clinically significant conditions such as anemia, end stage renal failure, myocardial ischemia, cerebrovascular thrombosis, ongoing anticoagulant therapy or presence of a bleeding diathesis or history of thrombotic events.

Randomization was performed using sequential numbers. The case group (n=100) received IV TA (15 mg/kg) and the control group (n=70) received normal saline in identical syringes. Both participants and study staff (site investigators and trial coordinating center staff) were blinded to treatment allocation.

Intraoperative hemorrhage was estimated by the attending anesthesiologist at the end of surgery by accounting for loss of blood and irrigation fluid in a 25-mL-graded suction canister and nasopharyngeal packing (measured weight of packing on the electronic scale). Moreover, at the end of a surgery, the surgical field was graded in terms of bleeding by the surgeon. 

Hemodynamic parameters, including systolic and diastolic arterial blood pressure (BP), and heart rate (HR) were recorded at 15-minute intervals. Prothrombin time, partial thromboplastin time, and complete blood count were measured before surgery and 6 hours postoperatively. The occurrence of possible side effects of treatment such as nausea, vomiting, pain and epistaxis were evaluated in the post-anesthesia care unit (PACU). Moreover, pain after surgery was measured on a visual analog scale (VAS).

*Statistical analysis*

Data are presented as means (standard deviation), medians (ranges), or percentages, as appropriate. Baseline characteristics of the two groups were analyzed using Student's t-test for continuous data and the Chi-square test for categorical analysis. Repeated measures of BP and HR were analyzed using repeated measures analysis of variance (ANOVA). Ranked data, including bleeding, were compared between groups using the Mann-Whitney U test. All comparisons were two-tailed. P<0.05 were considered statistically significant. Statistical analyses were performed using SPSS version 20.00 software (SPSS, Inc., Chicago, IL,USA).

## Results

In total, 170 patients (90 male [53%] and 80 female [47%]) with a mean age 30.54±4.14 years were evaluated (Table 1). The correlation between demographic characteristics such as sex and age with blood loss and TA efficacy was not significant (P>0.05).

Bleeding volume was significantly lower in the TA group compared with the placebo group(107.7±45.1 vs 189.3±51ml; P<0.001).

There was no significant difference between pre- and postoperative hematocrit (38.81±4.20 vs. 36.60±3.35) or hemoglobin level (12.51±2.5 vs. 11.64±1.9) in the TA group (P>0.05 in both cases). Moreover, the difference between the TA and control groups regarding postoperative hematocrit level (34.65±4.45 vs. 36.60±3.35) and hemoglobin (10.81±2.1vs. 11.64±1.9) was not significant (P>0.05).

Vomiting and nausea in the control group was greater than in the control group, but the difference was not significant (P>0.05). Clinical evidence of arterial or venous thrombosis was not detected in any of our patients.

Moreover we did not detect significant coagulation alterations in the TA group. Additionally epistaxis was not detected in any patients (Tables. 1-3).

**Table 1:** Demographic characteristics of patients

		**Tranexamic Acid (N=100)**	**Control** **(N=70)**	**P-value**
Gender	Male	45 (45%)	40 (57%)	0.6
	Female	55 (55%)	35 (50%)	
Age (Mean ± SD)		32.4±3.24	29.7±4.32	0.7

**Table 2:** Hemodynamic evaluation of patients in two groups

**Groups**	**Control**	**Tranexamic**
Baseline SBP (mmHg)	116.95 ± 12.05	111.80 ± 8.99
Baseline DBP (mmHg)	73.13 ± 7.12	72.11 ± 6.31
Pulse (beats/min)	76.30 ± 11.40	79.85 ± 13.58
Preoperative hematocrit	(%) 39.47 ± 3.89	(%)38.81 ± 4.20
Postoperative hematocrit (at 6 h)	(%)34.65 ± 4.45	(%)36.60 ± 3.35
Preoperative hemoglobin	12.31 ± 2.2	12.51 ± 2.5
Postoperative hemoglobin (at 6 h)	10.81 ± 2.1	11.64 ± 1.9
Intraoperative SBP (mmHg) (Mean of tree times measurements)	110.13 ± 11.23	111.00 ± 8.87
Intraoperative DBP (mmHg) (Mean of tree times measurements)	72.11 ± 6.56	70.85 ± 9.34
Intraoperative pulse (beats/min) (Mean of three measurements)	74.28 ± 10.23	73.45 ± 9.87
Postoperative SBP (mmHg) (Mean of three measurements)	107.11 ± 10.23	109.11 ± 7.97
Postoperative DBP (mmHg) (Mean of three measurements)	70.13 ± 6.50	71.12 ± 8.74
Postoperative pulse (beats/min) (Mean of three measurements)	73.22 ± 10.12	72.33 ± 8.17

DBP: diastolic blood pressure; SBP: systolic blood pressure

**Table 3:** Postoperative comparison of two groups

**Groups **	**Placebo**	**Tranexamic Acid**	**P-value**
INR	1.1 ± 0.1	1.1 ± 0.2	0.3
PTT	31.6 ± 2.4	27.8 ± 11.6	0.02
PT	10.5 ± 0.76	11.3 ± 1.2	0.052
Platelets	2,78×10^5 ^± 10700	2.63×10^5 ^± 94000	0.5
Blood loss	189.3 ± 51.2	107.7 ± 45.1	0.001
VSA	4.1 ± 0.75	4.5 ± 1.46	0.3
Hospital stay duration	4.3 ± 0.72	4.9 ± 1.7	0.3
Lund score	15.31 ± 4.56	17.32 ± 4.51	0.8


## Discussion

Reducing hemorrhage and increasing visibility of the surgical field are two major goals for surgeons to improve the outcome of endoscopic sinus surgery (1–7). TA is a synthetic derivative of the amino acid lysine that blocks the lysine- binding sites on plasminogen molecules reversibly. TA competitively inhibits activation of plasminogen thereby reducing conversion of plasminogen to plasmin, an enzyme that degrades fibrin clot, fibrinogen, and other plasma proteins, such as procoagulant factors V and VIII (1-5). Previous studies proved that TA is effective when administered via the topical and IV route (4,9-14,16). 

In this study, administration of IV TA (15 mg/kg) resulted in a significantly lower intraoperative bleeding volume compared with the placebo group, with no thrombotic events or alterations in coagulation parameters postoperatively. Our finding was consistent with a study by Chhapola et al. that evaluated the effect of TA in 200 patients undergoing endoscopic nasal surgery and showed that intraoperative hemorrhage decreased (72%) in patients who received preoperative TA. Moreover, similar to our findings, they reported no posto- perative alteration in coagulation parameters (13). In line with our experience, another randomized, double-blinded, controlled trial study by Alemian et al. evaluated the effects of IV TA (10 mg/kg) on hemorrhage and surgical field quality during FESS. This study indicated that IV TA effectively reduced bleeding and improved the surgical field during FESS (14). In contrast, in a randomized controlled trial of 40 patients, Kaewpradub et al. demonstrated that 5% TA in an irrigant fluid during orthognathic surgery did not significantly decrease intraoperative blood loss compared with a placebo group (15). A possible explanation for such discrepancy among reported studies is differences in experimental design and the technique used in ESS surgery as well as different doses and different routes of administration.

The most common early postoperative side effects of TA are nausea, vomiting and thrombotic events (4). The current study revealed no thrombotic events; however we revealed rates of nausea and vomiting of 7% and 12% in the TA and placebo groups, respectively, with no significant difference between the two groups. Similarly, Alemian et al reported less than 15% nausea and vomiting in their study and showed no thrombotic events in their patients. In addition, although risk of thrombosis is theoretically possible, it has not been proved in prior surveys (15). 

Although the administration of TA decreased blood loss in the current experience, it did not affect the postoperative levels of hemoglobin and hematocrit. This is consistent with the findings of a meta-analysis performed by Song et al. which showed that while IV administration of TA can effectively reduce intraoperative blood loss in orthognathic surgery, it did not affect postoperative levels of hemoglobin and hematocrit (16). It seems that TA has further beneficial effects; for example Ker et al in a meta-analysis demonstrated that TA reduces blood transfusion and mortality rates in patients undergoing surgery (17). 

One of the greatest problems in ESS is poor field visibility. Chhapola et al. showed that TA improves visibility of the surgical field and increases surgeon satisfaction (13). 

The main limitations of our study were the relatively small sample size and short postoperative duration of follow-up. Further investigations are recommended with longer postoperative follow-up periods and larger sample sizes to validate the findings reported here.

## Conclusion

We demonstrated that TA significantly decreased blood loss without an increase in important side effects such as alteration of coagulation parameters, hemodynamic changes, or vomiting and nausea. Furthermore, administration of TA means that anesthesiologists do not need to use antihypertensive agents in order to reduce blood loss in ESS.
